# Tyrotoxicon, Its Presence in Cheese, Ice Cream and Milk, and Its Relations to Cholera-Infantum

**Published:** 1888-01

**Authors:** V. C. Vaughan


### TYROTOXICON, ITS PRESENCE IN CHEESE, ICE CREAM AND MILK, AND ITS RELATIONS TO CHOLERA-INFANTUM

BY V. C. VAUGHAN, M. D.

It has long been known that the eating of cheese is sometimes followed by toxic symptoms. Instances of this kind have frequently been observed both in this country and in Europe. The symptoms produced are dryness and constriction of the throat, nausea, retching, vomiting, purging, and nervous prostration. Many German chemists have endeavored to isolate the poisonous principle, and many conjectures concerning it have been offered. However, all of these have been unsatisfactory, as is evidenced by the following quotations : Dr. Arnold Hiller says : "Nothing definite is known of the nature of cheese-poison," Die Lehre der Gaulniss, Berlin, 1879, p. 197. Professor Husemann states : "The older investigation of the chemical nature of cheese-poison, which led to the belief of putrefaction, cheese-acids, and other problematic substances are void of all trustworthiness, and the discovery of the active principle of cheese-poisoning may not be looked for in the near future, on account of the want of proper animals for controlling the experiment with the extract, as dogs can eat large quantities of cheese without producing any effect." Real-Enclyclofœdia der gessammten Heilkunde, Berlin, 1881, Vol. VII, page 313.

During the year 1883 and '84, there were reported to the Michigan State Board of Health some 300 cases of cheese-poisoning. All of these were caused by eating of twelve different cheeses. Of these, nine were made at one factory and one at three other factories. I received larger or smaller samples of each cheese for analysis. Of two I received about 38 pounds each. After many months of experimentation, I succeeded in isolating a poison from this cheese. The method adopted was as follows : An aqueous extract of the cheese was made, and filtered through heavy Swedish filter paper. The filtrate, which was strongly acid, was rendered slightly alkaline and then agitated with ether. After separation the ether was removed with a pipette and allowed to evaporate spontaneously. The residue was dissolved in distilled water and again agitated with ether. The spontaneous evaporation of this portion of ether left the poison in a pure cystalline form.

I have named this substance tyrotoxicon (cheese-poison.) It gives with potassium, ferricyanide and ferric chloride, Prussian blue. It also reduces iodic acid. The ordinary alkaloidal reagents fail to precipitate it. The crystals have a penetrating old cheesy odor, and it is interesting in this connection to state that Husemann and Bœhm have both observed this odor in poisoning sausage. If the crystals be allowed to stand at ordinary temperature they decompose with the formation of an organic acid, the nature of which has not been determined.

A few drops of an aqueous solution of these crystals placed upon the tongue produces all the symptoms observed in those who had been made sick by eating of the cheese. This was tried repeatedly upon myself and upon some of my students who kindly offered themselves for experimentation.

In November, 1885, a student brought me a four-ounce bottle partly filled with milk which had stood tightly closed with a glass stopper for about six months. From this I succeeded in obtaining a trace of the poison.

Afterwards, I obtained from a gallon of milk which had stood for three months in a closed bottle, some of the crystals. Ten drops of an aqueous solution of these crystals placed in the mouth of a small dog, three weeks old, caused, within a few minutes, frothing at the mouth, retching, the vomiting of a frothy fluid, muscular spasms over the abdomen, and after some hours watery stools. The next day the dog seemed to have partially recovered but was unable to retain any food. This condition continuing for two or three days, the animal was killed with chloroform. No examination of the stomach was made.

This experiment with the dog shows that the lower animals are subject to the influence of the poison, and the only reason why no symptoms have been produced in cats and dogs by feeding them cheese is that in the quantity of cheese which they will eat they do not get enough of the poison to affect them.

June 13, 1886, I received from Dr. Henry B. Baker, Secretary of the Michigan State Board of Health, a pint bottle about two-thirds full of melted ice cream, with the request that I analyze it, as some eighteen persons had been seriously affected by eating of it. Dr. Baker had also sent some of the vanilla which had been used as flavoring. It was thought that the poison had been found in the vanilla, as some lemon cream, furnished by the same caterer, had not affected those who ate of it. As the readiest and most positive means of deciding it, my assistant, Mr. Novie, and myself took at first thirty drops each of the vanilla extract. No ill effects following this, Mr. Novie took two teaspoonfuls more, with no results. This settled the question of the supposed poisonous nature of the vanilla.

From this ice-cream, proceding as with the cheese, I obtained the crystals of tyrotoxicon, and with them produced nausea, vomiting, and diarrhœa in a cat.

After twenty-four hours these symptoms in the cat had subsided, but it was still unable to eat anything. Three days later the animal was placed under the influence of ether and its abdomen was opened. We certainly expected to find marked inflammation of the stomach. But we really did find the stomach and small intestines filled with a frothy, serous fluid, such as had formed the vomited matter, and the mucus membrane very white and soft.

There was not the slightest redness anywhere. The liver and other abdominal organs seemed to be normal It may be remarked that this condition of the cat's stomach corresponds with that so often observed in children after death from cholera-infantum.

The custard of which this cream was made was allowed to stand in a very foul atmosphere, so I was informed by a resident of the place in which the caterer lived, some two hours before it was frozen. During this time fermentation was undoubtedly going on.

By placing small bits of the poisonous cream in good milk, allowing it to stand for twenty-four hours, the whole becomes poisonous. This proves that the poison is due to some ferment. Recently, Drs. W. K. Newton and Shippen Wallace, analysts for the New Jersey State Board of Health, have made an important contribution to our knowledge of tyrotoxicon. Their report may be found in the Philadelphia *Medical News*, of September 25, 1886. Many persons at different hotels at Long Branch were poisoned by milk. The poisonous milk was obtained from one milkman. Drs. Newton and Wallace found that the cows were milked at unusual hours of midnight and noon. The noon milking was immediately placed in cans, without being cooled, and "carted eight miles during the warmest part of the day in a very hot month." It was the milk which produced the poisonous effects. The morning's milk was always good.

To the medical profession the most interesting point connected with this poison is its probable relation to cholera-infantum. There is great similarity between the symptoms produced by the poison and those observed in the disease. The suddenness and violence of the attack, the nausea and vomiting without marked tenderness of the abdomen, the great thirst, the severe pain in the back of the head, the nervous prostration and the tendency to deep sleep are observed in both. Again, the white soggy appearance of the mucus membrane of the cat corresponds exactly with observations in children after death from cholera-infantum. Cholera-infantum, as is stated by Smith, "is a disease of the summer months, and with the exceptional cases of the cities."

Thus the disease occurs at a time when decomposition of milk takes place most rapidly. It occurs at places where the absolutely fresh milk often cannot be obtained. It is most prevalent among people whose surroundings are most favorable to fermentative changes. It is most fatal at an age when there is the greatest dependence upon milk as a food, and when, on account of the rapid development of the intestinal follicles, there is the greatest susceptibility to the action of an irritant poison, and when irritation and nervous fevers are most easily induced.

The following case will illustrate the relation between cholera-infantum and tyrotoxicon:

July 30, 1886, about one o'clock, P. M., I was called to see the seven months old babe of Mr. B. I found that the child had been vomiting quite constantly for about three hours. It had also passed watery stools some six or seven times. The eyes were sunken, skin cold and clammy, and pulse rapid and small. I diagnosed cholera infantum. During the preceding night the child had seemed as well as usual, and had taken nourishment freely from the mother's breast. Early in the morning it had been given a bottle of cow's milk, and soon thereafter the nausea began. Later, as stated above, the child began to purge. The mother furnishing an insufficient supply of milk, it had been the habit to give the child cow's milk several times during the day. I prohibited the further use of milk, both that from the mother and from the bottle, and substituted meat preparations and rice-water as foods. I also prescribed pepsin, bismuth subnitrate, chalk mixture, and camphorated tincture of opium.

The cow's milk which had been furnished the child was from an animal kept by one of the neighbors. On the evening of the same day that the child was taken sick, I obtained two quarts of the morning's milk of this animal. The milk had the appearance of very rich cream, being of a yellow tint throughout. This milk was allowed to stand through the night of the 30th in the ice-box of a refrigerator. On the morning of the 31st I began the analysis. After pouring the milk from the pitcher, there remained in that vessel about two ounces of a fluid the color of port wine. Microscopical examination of this fluid showed the presence of pus and blood corpuscles. The blood was also detected by obtaining the characteristic bands of oxyhemoglobine with the spectroscope. The milk, which had already coadgulated, was filtered. The strongly acid filtrate was rendered feebly alkaline with potassium hydrate, and then agitated with absolute ether. After separation the ether was removed with a pipette and allowed to evaporate spontaneously. The residue was dissolved in distilled water and again agitated with ether. The etheral solution left after spontaneous evaporation a residue which had a slightly brownish tint. I did not obtain the crystals of tyrotoxicon, doubtless owing to this trace of impurity, but the residue had the odor and taste of tyrotoxicon. The residue dissolved in some distilled water and given to a cat produced retching and vomiting.

That tyrotoxicon was present in the milk taken by the child shortly before the beginning of its illness, there could be no doubt. It is true that the milk was abnormal in other respects also, inasmuch as it contained pus and blood.

After the withdrawal of all milk and the use of the medicinal agents mentioned above, the child began to improve, and by the afternoon of August 1, it seemed so well that it was allowed a bottle of good cow's milk (from another animal); but soon after taking this milk it again began to vomit and purge. Milk was again withheld and the same medical treatment was resorted to. This attack was slight, and after it the child continued to improve until the night of August 4th, when the grandmother, "who knew more about nursing babies than the doctor," fed the child bountifully upon milk. Again the vomiting and purging began, and it was more than a week before all symptoms had disappeared. About the 15th of August, milk was again allowed ; at first in small quantity, and this seeming to have no harmful effect, more liberal quantities were given. The child has continued well ever since.

From the above observed facts I infer that not only the poison but the ferment by whose growth the poison is generated was introduced to the alimentary canal, that this micro-organism continued to live until some time after August 4th, and when the milk was given, the poison was again formed by the growth of the ferment. Whether or not any germicide could have been borne by the child in sufficient quantity to destroy the ferment I do not know.

It now remains to ascertain with certainty the nature of the ferment concerned in the production of tyrotoxicon, and to determine by experiments upon milk inoculated with this germ the value of various germicides. Many physicians claim that the bichloride of mercury in proper doses is a very valuable agent in the treatment of cholera-infantum.

Fortunately, no other children were furnished with the milk of this cow which first supplied the baby. The attention of the owner of the cow was called to the nature of the milk, and its use by all was discontinued for some days. There was no sore visible upon the teats. Had this milk been mixed with that of a number of other animals the color would have escaped detection, and all the milk might been rendered poisonous.—*Journal of Reconstructives.*

